# Analysis of fetal dose exposure by modern radiation therapy in pregnant patients with supradiaphragmatic Hodgkin lymphoma—a phantom-based simulation

**DOI:** 10.1007/s00066-025-02440-w

**Published:** 2025-08-01

**Authors:** Gina M. Smeets, Isabel Vogt, Heidi Wolters, Christopher Kittel, Dominik A. Hering, Fabian M. Troschel, Gabriele Reinartz, Burkhard Greve, Uwe Haverkamp, Michael Oertel, Hans T. Eich

**Affiliations:** Department of Radiation Oncology, University Hospital Muenster, Muenster, Germany

**Keywords:** Radiation oncology, Pregnancy, Involved-site radiotherapy, Toxicity, Side effects

## Abstract

**Purpose:**

Modern involved-site radiotherapy (ISRT) for Hodgkin lymphoma decreases toxicity through reduced field sizes and radiation doses. However, in pregnancy, the therapeutic benefit has to be weighed against putative harm to the mother and the fetus, as even small doses may have deleterious effects. We conducted a phantom-based simulation to analyze uterine dose exposure from cervical and mediastinal ISRT.

**Methods:**

Cervical and mediastinal ISRT target volumes were contoured to calculate three comparison plans (3D-conformal radiotherapy [3D-CRT], intensity-modulated radiotherapy [IMRT], volumetric intensity-modulated arc therapy [VMAT]). Thermoluminescent dosimeters (TLD) were placed within a humanoid Alderson phantom to simulate early and late pregnancy. Overall, six measurements (two for every radiotherapy plan) with 38 TLD were conducted.

**Results:**

In early pregnancy, cervical ISRT treatment of 19.8 Gray (Gy) resulted in median fetal exposures of 8.8 mGy, 15.4 mGy, and 9.9 mGy for 3D-CRT, IMRT, and VMAT, respectively, with significant differences between the three techniques (*p* < 0.001) and increased doses in late pregnancy (*p* < 0.001). For mediastinal ISRT (19.8 Gy), early pregnancy doses were 44 mGy, 63.8 mGy, and 60.5 mGy for 3D-CRT, IMRT, and VMAT, respectively, again with significant differences (*p* < 0.001) and a significant increase (*p* < 0.001) in late pregnancy. In comparison, values of 214.2 mGy (3D-CRT), 249.9 mGy (IMRT), and 249.9 mGy (VMAT) were reached using 30.6 Gy, with significant differences between 3D-CRT and VMAT (*p* < 0.001), 3D-CRT and IMRT (*p* < 0.001), and IMRT and VMAT (*p* = 0.004).

**Conclusion:**

Using RT during pregnancy may have deleterious effects on the fetus and should be deferred until after birth whenever possible. Theoretical uterine RT doses are low overall and only exceeded safety thresholds with higher-dose intensity-modulated plans. The indication for RT in pregnancy always requires careful risk–benefit consideration and individualized planning.

## Introduction

Malignant diseases in pregnant women are rare, with an overall incidence of 1 in 1000 pregnancies [1, 2]. With an incidence of 0.016–0.1%, lymphomas constitute some of the most common malignancies in this setting [3–5]. Radiotherapy (RT) is an efficient and important treatment modality for lymphoma, using limited doses and field designs in the modern era [6, 7]. Nevertheless, it remains uncertain whether this effective treatment can be safely administered in pregnant women. There is no guideline on this rare treatment scenario and, therefore, the decision must be weighed and evaluated individually for each patient. For a long time, the recommendation was to postpone treatment until after birth or to advise terminating the pregnancy to initiate therapy as quickly as possible, instead of risking a malformation or impaired fetal development [8, 9]. Thus, on an ethical level, the health and therapeutic benefits for the mother have to be carefully balanced against the potential risks and side effects in the fetus [10].

With the implementation of new technical and conceptual innovations in radiation oncology, it is tempting to speculate whether treatment of the maternal malignancy and protection of fetal development can be achieved at the same time [6, 7]. Therefore, a phantom-based analysis was undertaken to study the dose exposure of a hypothetical fetus during state-of-the-art lymphoma irradiation at different stages of pregnancy. The study investigates the impact of different irradiation techniques for cervical and mediastinal target volumes and attempts to test for treatment safety. It further illustrates the complicated landscape of radio-oncological treatment during pregnancy by providing a selective review of the existing literature.

## Materials and methods

### Alderson radiotherapy phantom

**Fig. 1:**
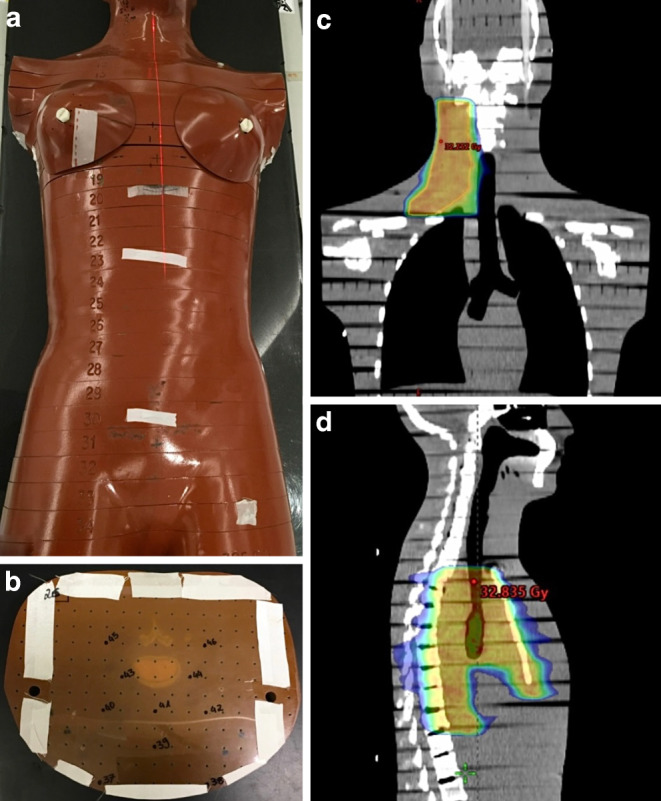
Radiotherapy planning and simulation. **a** Alderson phantom in planning position during CT scan. **b** Representative thermoluminescent detector positions in the upper abdominal layers 23–26 representing late stages of pregnancy (25–36 weeks of pregnancy). **c** Cervical target volume in coronal plane. **d** Mediastinal target volume in sagittal plane including the upper + lower mediastinum. **c**, **d** Contouring of the target volumes showing the 95% isodose in color-wash visualization

An Alderson radiotherapy phantom (ART) was used to simulate fetal dose exposure in pregnant patients with lymphoma. The ART is a reproduction of soft tissue, bones, and lungs and has density (ρ = 0.985 g/cm^3^) [11], absorption, and scattering properties equivalent to those of the human body. With a length of 155 cm and a weight of 50 kg, the phantom represents the female body without extremities and is separated into a total of 34 horizontal slices of 2.5 cm (Fig. 1a). The individual slices contain cavities with a distance of 1.5 cm into which thermoluminescent detectors (TLD) can be inserted (Fig. 1b).

### Treatment planning system/radiotherapy

For RT planning, a CT of the ART was acquired with a slice thickness of 3 mm using a Toshiba Aquilion machine (Toshiba, Tokio, Japan). Contouring and planning were performed in the Eclipse treatment system, version 15.6 (Siemens Healthineers, Erlangen, Germany) [12]. Organs at risk in the cervical and mediastinal regions were delineated, and one prototypical involved-site target volume each for the right neck and the mediastinum was defined following state-of-the-art guidelines (Fig. 1c, d; [6, 13]). For the cervical target volume, lymph node involvement of the upper and lower neck was assumed, whereas for the mediastinal target volume, both the upper and lower mediastinum were defined as involved regions. The lower border of the mediastinal target volume was the base plate of thoracic vertebra 10, just above the diaphragm. As the ART does not account for pathological anatomy such as lymph node expansion, the simulated patient was supposed to be in complete remission (after systemic therapy). An isotropic expansion from the clinical to the planning target volume of 1 cm was applied. For each target volume, three radiation plans were created: 3D-conformal RT (3D-CRT), intensity-modulated RT (IMRT), and volumetric intensity-modulated arc therapy (VMAT), resulting in a total of six plans. The planning templates used a daily fraction dose of 1.8 Gray (Gy) and a photon energy of 6 MV to avoid secondary neutrons.

In all six radiation plans, a dose of 19.8 or 30.6 Gy in single fractions of 1.8 Gy was prescribed to the planning target volume following International Commission on Radiation Units and Measurements reports 50 and 62 [14–16]. Five irradiation fields were used for 3D-CRT: three fields with an enhanced dynamic wedge (EDW) and two without, resulting in a total of 221 monitor units (MU). Likewise, the cervical IMRT-plan utilized five fields with 548 MU. For VMAT planning, a half-side rotation was used, starting counterclockwise from 0–181° and continuing clockwise from 181–0° with 310 MU. In comparison, mediastinal 3D planning used four fields (two with and without EDW) and 263 MU. Mediastinal IMRT was planned with six fields and 1140 MU and mediastinal VMAT via two full rotations using 639 MU.

### Thermoluminescent detectors and measurements

Thermoluminescent detectors (TLD) TLD-100H (ThermoFisher Scientific GmbH, Waltham, MA, USA) with dimensions of 1 × 1 × 6 mm were used for simulation, with a total of 38 TLD placed within the ART for each measurement. Nine additional TLD were positioned in a 1.3 cm thick Plexiglas plate as a control standard and irradiated with a single dose of 2 Gy as reference. To simulate early stages of pregnancy (weeks 12–24), the lower abdominal layers 27–30 were taken into account, whereas for later stages of pregnancy (weeks 25–36), upper layers 23–26 were considered (Fig. 1b).

After radiation treatment, the TLD were heated and the photon energy emitted was measured on a heating plate using the Harshaw Bicron Model 5500 Automatic TLD Reader (ThermoFisher Scientific GmbH, Waltham, MA, USA) and the TLDSHELL.EXE software, in which a glow curve is displayed for each individual TLD. The emitted photon energy is proportional to the previously absorbed energy. After measurements, TLD were regenerated by heating to 400 °C for 15 min in the TLD annealing oven type TLD-Heat (RadPro International GmbH, Remscheid, Germany).

The absolute values were then extrapolated to calculate the total treatment exposure of 19.8 and 30.6 Gy as standard doses for early-stage favorable and unfavorable Hodgkin lymphoma, respectively [17], referring specifically to the medians, means, standard deviations, and one-sided *p*-values.

## Results

### Cervical—early pregnancy (weeks 12–24)

**Fig. 2:**
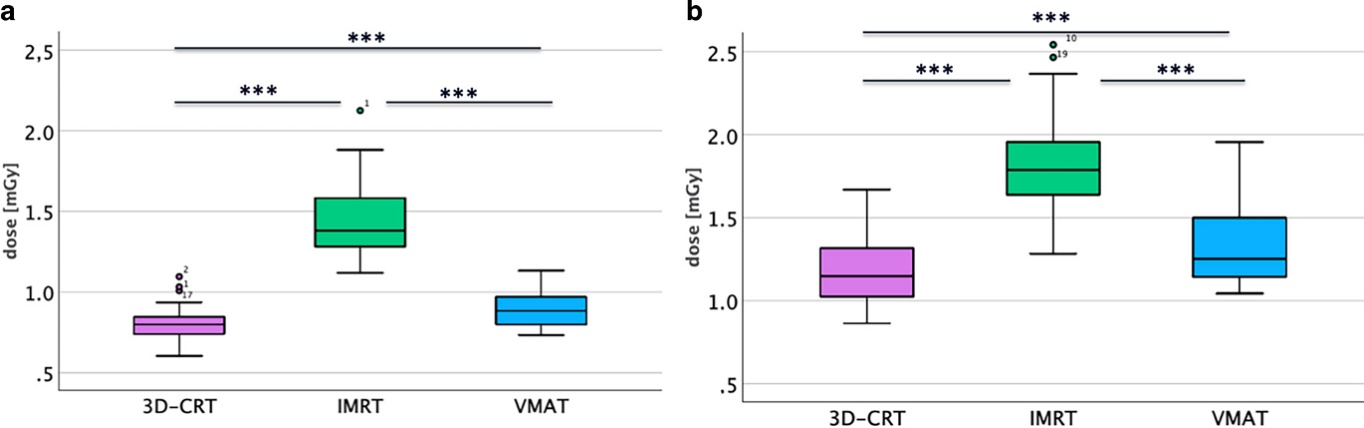
Dose exposure to the uterus for cervical involved-site radiotherapy. **a** Dose per fraction in mGy for early pregnancy using 3D-CRT, IMRT, and VMAT. **b** Dose per fraction in mGy for late pregnancy using 3D-CRT, IMRT, and VMAT. Abbreviations: 3D-CRT: 3D-conformal radiotherapy, Gy: Gray, IMRT: intensity-modulated radiotherapy, VMAT: volumetric intensity-modulated arc therapy

In early pregnancy, a fraction dose of 1.8 Gy resulted in a median fetal exposure of 0.8 mGy (range: 0.6–1.1 mGy) when using 3D-CRT, 1.4 mGy (range: 1.1–2.1 mGy) for IMRT, and 0.9 mGy (range: 0.7–1.1 mGy) for VMAT (Fig. 2a), with significant differences between all three techniques (*p* < 0.001). Variability in the absorbed dose values was observed across all three techniques: in ascending order: VMAT (standard deviation: ± 0.10 mGy), 3D-CRT (standard deviation: ± 0.11 mGy), and IMRT (standard deviation: ± 0.22 mGy).

Assuming a total dose of 19.8 Gy, the median uterine exposure was 8.8 mGy for 3D-CRT, 15.4 mGy for IMRT, and 9.9 mGy for VMAT (Table 1). For a total dose of 30.6 Gy, the median uterine exposures increased to 13.6 mGy for 3D-CRT, 23.8 mGy for IMRT, and 15.3 mGy for VMAT (Table 1).

**Table 1:** Dose exposure to the cervical involved-site radiotherapy in mGy and mSv in early/late pregnancy. Abbreviations: 3D-CRT: 3D-conformal radiotherapy, Gy: Gray, IMRT: intensity-modulated radiotherapy, Sv: Sievert, VMAT: volumetric intensity-modulated arc therapy

Stage of pregnancy	Radiation technique	Radiotherapy dose (Gy)	Median uterus dose (mGy)	Mean uterus dose (mGy)	Weighting factor	Effective dose uterus (mSv)
Early	3D-CRT	19.8	8.8	8.8	0.05	0.44
		30.6	13.6	13.6	0.05	0.68
	IMRT	19.8	15.4	15.4	0.05	0.77
		30.6	23.8	23.8	0.05	1.19
	VMAT	19.8	9.9	9.9	0.05	0.49
		30.6	15.3	15.3	0.05	0.76
Late	3D-CRT	19.8	12.6	13.2	0.05	0.63
		30.6	19.5	20.4	0.05	0.97
	IMRT	19.8	19.7	19.8	0.05	0.99
		30.6	30.4	30.6	0.05	1.52
	VMAT	19.8	13.8	15.4	0.05	0.69
		30.6	21.4	23.8	0.05	1.07

### Cervical—late pregnancy (weeks 25–36)

In later stages of pregnancy, the median exposure per fraction of 1.8 Gy was 1.15 mGy (range: 0.9–1.7 mGy) for 3D-CRT, 1.80 mGy (range: 1.3–2.5 mGy) for IMRT, and 1.26 mGy (range: 1.0–2.0 mGy) for VMAT (Fig. 2b), with significant differences between all three techniques (*p* < 0.001). Dose variability increased across the techniques, with 3D-CRT showing the lowest variability (standard deviation: ± 0.18 mGy), followed by VMAT (standard deviation: ± 0.26 mGy) and IMRT (standard deviation: ± 0.29 mGy). Furthermore, the mean values of each individual technique differed significantly between early and later stages of pregnancy (*p* < 0.001).

In late pregnancy, a total dose of 19.8 Gy resulted in a median uterine exposure of 12.6 mGy for 3D-CRT, 19.7 mGy for IMRT, and 13.8 mGy for VMAT (Table 1). For a total dose of 30.6 Gy, the median uterine exposures increased to 19.5 mGy for 3D-CRT, 30.4 mGy for IMRT, and 21.4 mGy for VMAT (Table 1).

### Mediastinal—early pregnancy (weeks 12–24)

**Fig. 3:**
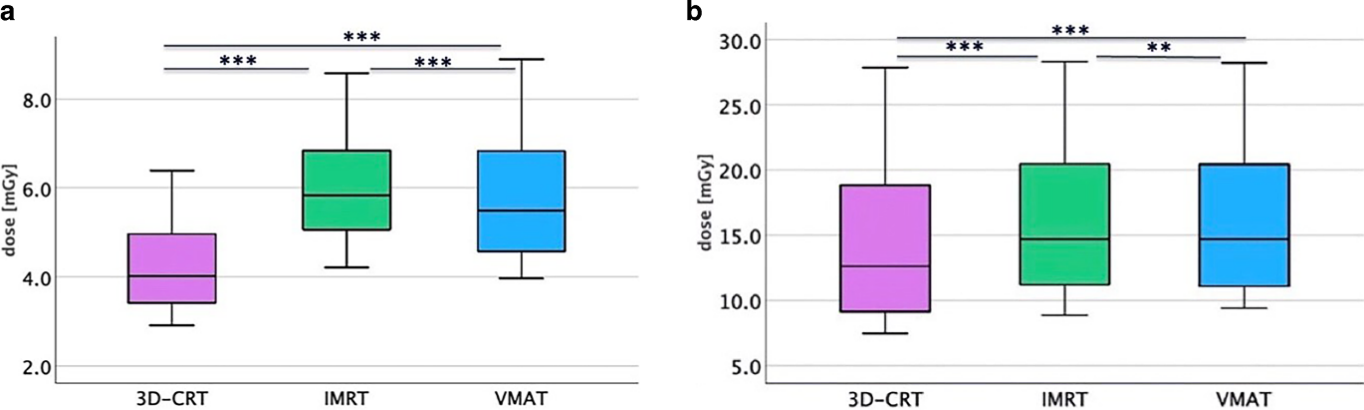
Dose exposure to the uterus for mediastinal involved-site radiotherapy. **a** Dose per fraction in mGy for early pregnancy using 3D-CRT, IMRT, and VMAT. **b** Dose per fraction in mGy for late pregnancy using 3D-CRT, IMRT, and VMAT. Abbreviations: 3D-CRT: 3D-conformal radiotherapy, Gy: Gray, IMRT: intensity-modulated radiotherapy, VMAT: volumetric intensity-modulated arc therapy

In the early phase of pregnancy, a fraction dose of 1.8 Gy resulted in a median fetal exposure of 4.0 mGy (range: 3.0–6.4 mGy) for 3D-CRT, 5.8 mGy (range: 4.2–8.6 mGy) for IMRT, and 5.5 mGy (range: 4.0–8.9 mGy) for VMAT (Fig. 3a), with significant differences between all three techniques (*p* < 0.001). Variability in absorbed dose values was observed across all three techniques: in ascending order: 3D-CRT (standard deviation: ± 1.13 mGy), IMRT (standard deviation: ± 1.35 mGy) and VMAT (standard deviation: ± 1.55 mGy).

Assuming a total dose of 19.8 Gy during early pregnancy, the median uterine exposure was 44 mGy for 3D-CRT, 63.8 mGy for IMRT, and 60.5 mGy for VMAT (Table 2). For a total dose of 30.6 Gy, the median uterine exposures increased to 68 mGy for 3D-CRT, 98.6 mGy for IMRT, and 93.5 mGy for VMAT (Table 2).

**Table 2:** Dose exposure to the mediastinal involved-site radiotherapy in mSv in early/late pregnancy. Abbreviations: 3D-CRT: 3D-conformal radiotherapy, Gy: Gray, IMRT: intensity-modulated radiotherapy, Sv: Sievert, VMAT: volumetric intensity-modulated arc therapy

Stage of pregnancy	Radiation technique	Radiotherapy dose (Gy)	Median uterus dose (mGy)	Mean uterus dose (mGy)	Weighting factor	Effective dose uterus (mSv)
Early	3D-CRT	19.8	44.0	49.5	0.05	2.2
		30.6	68.0	76.5	0.05	3.4
	IMRT	19.8	63.8	68.2	0.05	3.2
		30.6	98.6	105.4	0.05	4.9
	VMAT	19.8	60.5	66	0.05	3.0
		30.6	93.5	102	0.05	4.7
Late	3D-CRT	19.8	138.6	167.2	0.05	6.9
		30.6	214.2	258.4	0.05	10.7
	IMRT	19.8	161.7	184.8	0.05	8.1
		30.6	249.9	285.6	0.05	12.5
	VMAT	19.8	161.7	187	0.05	8.1
		30.6	249.9	289	0.05	12.5

### Mediastinal—late pregnancy (weeks 25–36)

In later stages of pregnancy, radiation exposure per fraction of 1.8 Gy was 12.6 mGy (range: 7.5–27.9 mGy) for 3D-CRT, 14.7 mGy (range: 8.8–28.3 mGy) for IMRT, and 14.7 mGy (range: 9.4–28.2 mGy) for VMAT (Fig. 3b). The mean values of 3D-CRT differed significantly from those of VMAT, with an average difference of 1.85 mGy (*p* < 0.001), compared to average differences of 1.61 (3D-CRT vs. IMRT) with *p* < 0.001 and 0.23 (IMRT vs. VMAT) with *p* = 0.004. In accordance with cervical RT, there were also significant differences between all techniques (*p* < 0.001 for 3D-CRT vs. VMAT or IMRT, respectively; *p* = 0.004 for IMRT vs. VMAT, with mean values of 16.8 mGy and 17.01 mGy) as well as significant differences between early and late pregnancy (*p* < 0.001).

Dose variability increased across the techniques, with IMRT showing the lowest variability (standard deviation: ± 5.79 mGy), followed by VMAT (standard deviation: ± 5.93 mGy) and 3D-CRT (standard deviation: ± 6.37 mGy).

A total dose of 19.8 Gy resulted in a median uterine exposure of 138.6 mGy for 3D-CRT, 161.7 mGy for IMRT, and 161.7 mGy for VMAT (Table 2). For a total dose of 30.6 Gy, the median uterine exposures increased to 214.2 mGy for 3D-CRT, 249.9 mGy for IMRT, and 249.9 mGy for VMAT (Table 2).

## Discussion

Treating lymphoma during pregnancy is complex, requiring careful consideration of both maternal and fetal health [10]. Since prospective randomized data on radiation exposure are not to be expected for ethical reasons and since the fetal risks of radiation exposure are derived primarily based on animal experiments [18] or survivors of nuclear disaster such as Chernobyl [19], phantom simulations are essential to provide valuable insights which may help guide clinical practice.

The standard treatment of classic Hodgkin lymphoma in early-stage disease consists of combined modality therapy with systemic treatment first followed by involved-site radiotherapy (ISRT) [20–22]. Polychemotherapy using doxorubicin, bleomycin, vinblastine, and dacarbazine (ABVD) may be safely administered in the second and third trimester [23]. According to recent first-line studies, this approach results in progression-free survival of 86–91% for patients achieving positron emission tomography (PET)-negative results after systemic therapy [24, 25]. For nodular lymphocyte-predominant Hodgkin lymphoma, RT alone has been shown to provide a favorable long-term outcome for patients with stage IA disease [26]. However, due to the indolent nature of disease, these patients may be observed first, with RT being deferred until after childbirth [27]. In summary, it is possible to postpone RT until after delivery [10, 28, 29] or even omit it based on an individual risk–benefit discussion. The latter may be an option for pregnant patients in complete remission, based on the excellent long-term results of chemotherapy alone (see above). It has to be kept in mind that the most effective protection is avoidance of RT during pregnancy, which, for Hodgkin lymphoma, is possible in most cases. The present simulation should not be misunderstood as a recommendation to initiate RT in pregnant patients but rather as a clinical biophysical risk estimation.

Both the mother and the unborn fetus are vulnerable to RT and must be protected optimally. The fetus is particularly sensitive to radiation in the early stages of pregnancy, such as the preimplantation phase, due to continuous cell division and differentiation [30]. Consequently, even small radiation doses during this stage can lead to severe consequences including fetal death [30, 31]. During the following organogenesis, malformations can result from radiation exposure of 0.1 Gy or more [31, 32]. In the period up to the 25th week of pregnancy, the central nervous system may also be affected by radiation exposure, with adverse effects including intellectual disability or cognitive impairments [30, 31, 33]. Radiotherapy may also increase the incidence of a secondary tumor for the child later in life [34]. As this risk is a stochastic effect with no lower threshold but a proportional increase with higher doses, any RT dose to the fetus may induce long-term side effects [10, 34]. These potentially devastating complications necessitate a very prudent indication of RT, avoiding it during pregnancy whenever possible. Despite these risks, previous studies have outlined the feasibility of irradiating the supradiaphragmatic neck and axillary and head fields in pregnant patients [3, 9]. To reduce the radiation dose to the fetus, protective lead blocks can be introduced [8, 10, 35, 36]. However, as these blocks have to be thick (and heavy) enough to protect from megavoltage radiation, mechanical injuries (e.g., from blocks falling on the mother’s abdomen) may occur—with drastic consequences for the fetus. Using a phantom-based simulation, we could demonstrate that the uterine exposure during modern cervical or mediastinal RT with 3D-CRT, IMRT, and VMAT is low overall, even without any external shielding devices, and may be considered acceptable under current guidelines [17, 37–41]. However, in a real-world application, additional shielding may be discussed to ensure optimal radiation protection.

In all radiation plans considered, the uterus and the fetus are outside the direct radiation field, although dose exposure increases with the duration of pregnancy and the growing size of the fetus, as expected according to the inverse-square law. Overall, this results in higher fetal dose exposure for the mediastinal target volume compared to the cervical target volume and, thus, in higher exposure during late pregnancy compared to early stages.

Comparing treatment techniques, IMRT frequently exhibits the highest dose among all the evaluated plans. When comparing IMRT to 3D-CRT, the fetal dose exposure is approximately 1.5 times higher on average. This increased exposure could be due to the requirement for at least twice as many MU in IMRT used for modulation to individual tumor shape, which is necessary to achieve conformal modulation of the radiation dose to irregular tumor shapes [37, 42].

Modern techniques such as IMRT and VMAT may achieve conformal target coverage [37–39], but they also create a higher low-dose exposure (“low-dose bath”) to larger areas of the body, which is suspected to increase the risk of secondary malignancies [38–40, 43, 44]. Consequently, 3D-CRT shows the lowest dose exposure and may be discussed as a possible treatment alternative in the setting of pregnancy.

In accordance, for the mediastinal target volume, the MU in IMRT were more than four times higher than those in 3D-CRT and nearly double those of VMAT. Despite still being significant, the differences in dose exposure between the radiation techniques were less pronounced for the mediastinal irradiation compared to the cervical ISRT. This suggests that the number of MU is outweighed by the impact of distance between the target volume and the uterus.

For the cervical target volume, only RT treatment with 30.6 Gy, as IMRT in early pregnancy or both IMRT/VMAT in late pregnancy, results in an effective equivalent dose exceeding the exposure limit of 1 mSievert (Sv) established for pregnant personnel according to German radiation protection laws [41]. In contrast, higher values are observed for the mediastinal target volume, with all three radiation techniques surpassing the exposure limit of 1 mSv during both early and late pregnancy. Although the exposure limit of 1 mSv is exceeded, none of the measured dose values for either the cervical or mediastinal target volumes approach the threshold of 20 mSv for prenatal radiation exposure defined for female patients by the German Society for Medical Physics [45]. In addition, the radiation exposure is below the threshold range of 50–100 mSv that is associated with deterministic cell and organ damage in the unborn child [46]. This indicates that higher radiation doses, particularly with intensity-modulated techniques, can lead to a significant increase in radiation exposure to the uterus but remain within acceptable thresholds according to current guidelines.

This analysis, based on a phantom simulation, has several limitations due to differences between human physiology and the ART configuration. Despite being an anthropomorphic model, the individual variability in size, weight, and stature in been women cannot be considered, which clearly indicates that the presented calculations cannot be applied to individual cases. Instead, in each case, the actual anatomy and size of the patient, the position and size of the fetus, and the treatment device and technique used are decisive for the actual fetal radiation exposure. In addition, the arrangement and size of internal organs in the phantom are standardized, which means that the anatomical changes during pregnancy, such as the size, volume, position, and shape of the uterus, are not fully captured. Therefore, the anatomical conditions of the mother during pregnancy are unique, as are the morphological and functional developmental differences of the fetus, such as the timing of organogenesis [37, 47–50]. This means that the values provided in this analysis may not be representative of the individual case. However, without the possibility of measuring directly within the patient, they may provide an idea of the magnitude of the actual numbers.

Moreover, the phantom can only approximate the radiation impact on the complex human physiology, as it may not account for metabolic processes, health conditions, individual radiation sensitivity, or immune responses. Additional limitations of the phantom simulation are that the measured values are reported as medians, meaning that maxima can potentially exceed the considered thresholds. Additionally, secondary scattered irradiation originating from the body is not accounted for, so the dose values given most likely underestimate the total dose to the fetus. Scattered radiation originates both from the linear accelerator head or the body tissue (internal component), the latter of which cannot be minimized by external shielding [51]. Total dose exposure (D_T_) to the uterus as well as its internal (D_in_) and external (D_ex_) components vary depending on the distance to the RT field and trimester of pregnancy [52, 53]. A previous study estimated the dose exposure for treatment of Hodgkin lymphoma at 83 mGy in the first trimester, with D_in_ and D_ex_ of 29 mGy and 54 mGy, respectively [51]. In advanced stages of pregnancy, the values are estimated to be significantly higher, with a D_T_ of 322 mGy, a D_in_ of 190 mGy, and a D_ex_ of 132 mGy, which demonstrates that the internal scattered radiation can account for over 50% of the total fetal radiation dose and illustrates the increasing scattered radiation resulting as the fetus grows and body volume increases [51]. The radiation technique also influences the level of scattered radiation; modern techniques such as IMRT require a higher number of MU, resulting in increased scattered radiation [44, 51, 54].

In addition to the uterus, other maternal risk organs have to be considered, particularly for the mediastinal irradiation with putative induction of cardiopulmonary toxicities, which can lead to an increased incidence of cardiac events as well as secondary malignancies after RT [55–60]. Key side effects of RT in the neck area involve mucosal changes [61] and alterations in the thyroid gland such as hypothyroidism [62]. Since the affected patients of childbearing age are usually young, the potential development of second malignancies after 5–10 years is a significant concern [57]. In a real-world setting, additional dose exposure from (staging) imaging will emerge: an fluorodeoxyglucose-PET scan, as a pivotal modality for staging and treatment planning assessment, would result in another 0.00616–0.0305 mGy/MBecquerel (total dose: 10mGy) [63], which is unacceptable in pregnant patients. Instead, staging via MRI without contrast agent is advised during pregnancy [31, 43, 64, 65]. Although recommended by the American College of Radiology independent of gestational age in the case of a clinical indication with no possibility of postponement until after birth [65], theoretical risks still remain. These include a potential temporary increase in maternal body temperature [66, 67] and possible teratogenic effects of the static magnetic fields on cell migration, cell differentiation, and cell proliferation [65]. In this context, the first trimester remains the most sensitive period for teratogenic influences [67]. However, without PET-CT there is no possibility of prognostic stratification, which is important, especially in intermediate-stage Hodgkin lymphoma [68].

The ISRT fields used in this simulation were based on fictional disease extents and are only exemplary for the individual patient. Due to the presumed involvement of a whole region, these fields have resemblance to conventional involved-field target volumes but use closer margins. In the future, limiting the RT to residual findings (comparable to advanced-stage disease) could lead to further dose reduction [69, 70], but this has not been tested in a clinical trial. This may be a promising approach to reducing uterine dose, especially for mediastinal disease, in later stages of pregnancy. In the present simulation, we decided to simulate “real-world” RT fields in order to obtain practice-orientated clinical data.

Other emerging advanced techniques such as modern active scanning proton therapy, particularly pencil-beam scanning, which were not evaluated in the present simulation, may offer further reductions in fetal dose exposure [71–74]. Pencil-beam scanning is accompanied by less scattered radiation in the accelerator, but it also induces the generation of secondary neutrons through proton interactions with tissue [74]. This dose component is not only difficult to quantify but also virtually impossible to shield, resulting in considerable uncertainty in precise dose estimation and limiting its use in pregnancy despite potential advantages [74]. Additionally, recent monocentric studies point towards the possible use of RT in the second or third trimester using lead shields [75, 76]. Treatment decisions were made based on a multidisciplinary approach in which trimester-specific treatment strategies were carefully considered to achieve the best possible outcomes for both mother and child [75, 76].

## Conclusion

Modern ISRT seems to offer the potential for low-risk RT of limited-stage supradiaphragmatic Hodgkin lymphoma during pregnancy. Still, the decision to treat (or not to treat) a patient with an oncological disease during pregnancy remains an individualized and carefully considered choice, weighing the risks and benefits of RT. Key factors to consider include the stage of pregnancy, progression, and the location of disease, as well as the distance of the radiation field to the fetus. In a real-life scenario, RT should be omitted during pregnancy or postponed until after birth. In the case of an urgent treatment situation, all measures like lead shielding (not applied here) and optimal planning should be applied. As the safest approach would be total omission of RT, each patient should be counseled by an interdisciplinary team of radiation oncologists, gynecologists, medical physicists, oncologists, and pediatricians. By doing this, individualized lymphoma treatment strategies during pregnancy, potentially including RT, may be feasible.
